# Bioactive Bacterial Nanocellulose Membranes Enriched with *Eucalyptus globulus* Labill. Leaves Aqueous Extract for Anti-Aging Skin Care Applications

**DOI:** 10.3390/ma15051982

**Published:** 2022-03-07

**Authors:** Tânia Almeida, Patrícia Moreira, Fábio J. Sousa, Cláudia Pereira, Armando J. D. Silvestre, Carla Vilela, Carmen S. R. Freire

**Affiliations:** 1 CICECO—Aveiro Institute of Materials, Department of Chemistry, University of Aveiro, 3810-193 Aveiro, Portugal; taniaralmeida@ua.pt (T.A.); armsil@ua.pt (A.J.D.S.); cvilela@ua.pt (C.V.); 2 CIBB—Center for Innovative Biomedicine and Biotechnology, University of Coimbra, 3004-504 Coimbra, Portugal; patriciaraquel_jm@hotmail.com (P.M.); fabio.fdti@hotmail.com (F.J.S.); cpereira@fmed.uc.pt (C.P.); 3 CNC—Center for Neuroscience and Cell Biology, University of Coimbra, 3004-504 Coimbra, Portugal; 4 CACC—Clinical Academic Center of Coimbra, 3004-561 Coimbra, Portugal; 5 Faculty of Pharmacy, University of Coimbra, 3000-548 Coimbra, Portugal; 6 Faculty of Medicine, University of Coimbra, 3000-548 Coimbra, Portugal

**Keywords:** bacterial nanocellulose, *Eucalyptus globulus* Labill. leaves, aqueous extract, antioxidant activity, sheet facial masks, anti-aging, skin care applications

## Abstract

Bacterial nanocellulose (BNC) membranes, with remarkable physical and mechanical properties, emerged as a versatile biopolymeric carrier of bioactive compounds for skin care applications. In this study, BNC membranes were loaded with glycerol (as plasticizer and humectant agent) and different doses (1–3 μg cm^−2^) of an aqueous extract obtained from the hydro-distillation of *Eucalyptus globulus* Labill. leaves (HDE), for application as sheet facial masks. All membranes are resistant and highly malleable at dry and wet states, with similar or even better mechanical properties than those of a commercial BNC mask. Moreover, the HDE was found to confer a dose-dependent antioxidant activity to pure BNC. Additionally, upon 3 months of storage at 22–25 °C and 52% relative humidity (RH) or at 40 °C and 75% RH, it was confirmed that the antioxidant activity and the macroscopic aspect of the membrane with 2 μg cm^−2^ of HDE were maintained. Membranes were also shown to be non-cytotoxic towards HaCaT and NIH/3T3 cells, and the membrane with 2 μg cm^−2^ of HDE caused a significant reduction in the senescence-associated β-galactosidase activity in NIH/3T3 cells. These findings suggest the suitability and potential of the obtained membranes as bioactive facial masks for anti-aging applications.

## 1. Introduction

The world population is considerably aged due to the decline in birth rate and the improvement in survival, and consequent increase in the average life expectancy, associated with the medical and technological developments achieved over the last decades [1,2]. The global population aged 60 or above is expected to reach about 2.1 billion by 2050, representing an increase of approximately twofold from 2017, and the number of people aged 80 or over is expected to nearly triple in the same period [3]. However, despite improved longevity, the aging of the skin is an inevitable process, and therefore, a growing demand for anti-aging skin care products is expected.

Skin aging is a complex biological process combining endogenous and exogenous mechanisms [4]. Endogenous aging is mainly determined by genetic factors and hormonal changes that occur with the normal aging process [4]. On the other hand, exogenous aging is mediated by external factors (e.g., overexposure to ultraviolet radiation (photoaging), gravity, smoking, pollution, and poor nutrition) [5]. In both aging processes, but particularly the exogenous one, it is well accepted that the increased oxidative stress, induced by reactive oxygen species (ROS) and resulting from the unbalance between ROS production and antioxidant defense, plays a crucial role [6,7,8]. With age, there is also a decrease in the ability of human skin cells to repair damaged DNA, which contributes to skin aging as well [8]. Thus, the aging process causes several biochemical alterations in skin composition, with effects in its structure and function [9], resulting in visible signs like wrinkling, dryness, loss of elasticity, thinning, rough texture, or irregular pigmentation [7,10]. Hence, with the growing pursuit for health and well-being, in which skin health and aesthetics are also included, the development of cosmetics incorporating bioactive compounds to prevent or attenuate skin aging and its external signs, thus improving skin appearance, has been at the forefront of research and innovation in the cosmetic industry over the last decades. In this context, and among cosmetics for topical treatment, beauty masks, and particularly sheet facial masks, are a growing market, with an expected compound annual growth rate (CAGR) of 8.1% from 2019 to 2027 [11]. This type of mask is especially attractive to modern consumers, largely due to its simple and easy application, fast use, and effectiveness [12]. Several materials can be used as the support matrix of these sheet masks, namely synthetic polymers (e.g., poly(vinyl alcohol) and silicone, fabrics (e.g., non-woven and cotton), hydrogels (e.g., collagen- and silk-based hydrogels) or cellulose nanofibers, such as bacterial nanocellulose (BNC) [13]. With the increasing demand for naturally derived skin care products, BNC has gained relevance and now stands as a superior bio-based material, already explored, and commercialized by some of the top cosmetics companies (e.g., Lancôme, Elizabeth Arden and DHC) [14] that are following the trend of green-oriented consumers’ demand.

Bacterial nanocellulose is an extracellular polysaccharide produced by several non-pathogenic bacteria from different genera (e.g., *Komagataeibacter* (formerly *Gluconacetobacter*), *Agrobacterium*, *Rhizobium*, *Escherichia*, and *Aerobacter*), through fermentation in the presence of oxygen and carbon sources (e.g., glucose) [15,16]. BNC is synthesized in the air–culture medium interface as an ultrafine 3D nanofiber network, showing high water content (>90%), high hydrophilicity, and a nanoporous structure [17]. When produced in static culture, BNC is obtained as a gelatinous hydrogel-like membrane with variable thickness and the shape of the culture vessel (viz. in situ moldability), which is an advantage when a predefined shape is desired [18]. The set of unique properties of BNC also embraces high crystallinity and purity, remarkable mechanical strength, and thermal stability [19]. Besides being biodegradable, BNC has also demonstrated good skin tolerance and biocompatibility in several in vivo studies [20,21,22], making it an ideal material for skin care products. In this regard, and because of its porous nanostructure, the applicability and effectiveness of BNC as carrier for the delivering of skin active compounds have been the focus of several works, for instance, for the delivery of caffein [23], silk-sericin [24], retinol [25], or rutin [26]. For further reading, a recent review provides a comprehensive overview of the versatility and applicability of BNC in green cosmetics, including as a carrier of skin active substances [27].

Natural bioactive compounds are widely used in anti-aging skin care cosmetics [28]. Plant extracts, in particular, are a rich source of bioactive compounds (e.g., polyphenols, terpenoids, vitamins, among others) with potential multiple actions on skin, with antioxidant and antimicrobial activities or tyrosinase inhibition effects being the main benefits reported so far [29]. Therefore, given the current trend for natural products in cosmetics, plant extracts appear among the most promising ingredients for the development of skin care products. Moreover, the incorporation of natural extracts into BNC membranes has also been investigated for cosmetic purposes. Examples are the works reporting the incorporation of propolis extract, known to have antiseptic and astringent activities [13,30], extracts from oat and rosemary with moisturizing effects [13], or the cosmetic formulation composed by *Adansonia digitata* fruit, *Hibiscus sabdariffa* flower, *Coffea arabica* seedcake, *Kigelia africana* fruit, and *Acacia* and *Crocus chrysanthus* bulb extracts, with anti-aging properties, namely wrinkle smoothing [21]. Recently, a review made a critical insight into the importance of associating BNC with plant phenolics to prevent UV-induced skin damage [31].

*Eucalyptus globulus* Labill. evergreen tree is a well-known source of bioactive compounds, namely phenolic compounds such as phenolic acids, flavonoids, or hydrolysable tannins [32,33,34,35,36]. The antioxidant and antimicrobial activities of *E. globulus* extracts containing these compounds have been demonstrated in several studies [34,35]. Concerning application in the cosmetic field, a recent study demonstrated the protective effect, both in vitro (in human dermal fibroblasts) and in vivo (hairless mice), of a 50% ethanolic extract from dried commercial *E. globulus* biomass against UV-induced photoaging, with promising results regarding the prevention of wrinkle formation and skin dryness [37]. However, to the best of our knowledge, *E. globulus* extracts have never been explored, in combination with a polymeric substrate, to produce bioactive skin masks for anti-aging skin care.

With this perspective, the purpose of the present work was to produce BNC membranes loaded with an aqueous extract derived from the hydro-distillation (hydro-distillation extract, HDE) of *E. globulus* leaves. Hydro-distillation is a method commonly used by the industry for the extraction of essential oils [38]. In addition, glycerol (G), a widely used humectant ingredient in cosmetics [39], was also incorporated in the BNC membranes (7.5 mg cm^−2^) to increase their flexibility and, at same time, the conformability to the skin. Thus, the goal of this work was to obtain an entirely bio-based material with functional properties for potential application as an anti-aging sheet facial mask. BNC membranes loaded with different doses of the hydro-distillation extract were characterized in terms of their morphology, mechanical properties, thermal stability, and moisture-uptake capacity. Moreover, the *in chemico* antioxidant activity of the different membranes was also evaluated, as well as their in vitro cytotoxicity (in human keratinocytes and mouse fibroblasts cell lines). Additionally, the antioxidant activity stability after 3 months of storage, in the dark, at 22–25 °C and 52% relative humidity (RH) or at 40 °C and 75% RH of the membrane with 2 μg cm^−2^ of HDE was assessed. Finally, the in vitro anti-senescence activity of the BNC membrane with 2 μg cm^−2^ of HDE was further investigated.

## 2. Materials and Methods

### 2.1. Chemicals and Materials

Citric acid (≥99.5%), dimethyl sulfoxide (DMSO) (≥99.0%), disodium hydrogen phosphate (≥99.0%), glucose (≥99.5%), glycerol (≥99.5%), magnesium nitrate hexahydrate (≥99.0%), potassium chloride (≥99.0%), potassium dihydrogen phosphate (≥99.0%), potassium sulphate (≥99.0%), sodium bicarbonate (≥99.5%), sodium chloride (≥99.0%), and 3-(4,5-dimethylthiazol-2-yl)-2,5-diphenyltetrazolium bromide (MTT) (≥97.5%) were purchased from Sigma-Aldrich (Lisbon, Portugal). All other chemicals were of laboratory grade. Calcium chloride, Dulbecco’s Modified Eagle’s Medium (DMEM), etoposide, Folin–Ciocalteu’s phenol reagent, magnesium chloride, sodium pyruvate, trypsin-EDTA solution, N-(2-hydroxyethyl)piperazine-N′-(2-ethanesulfonic acid), 4-(2-hydroxyethyl)piperazine-1-ethanesulfonic acid (HEPES), and 2,2-diphenyl-1-picrylhydrazyl (DPPH) were supplied by Sigma-Aldrich (Lisbon, Portugal). Peptone and yeast extract were acquired from Himedia Laboratories GmbH (Einhausen, Germany). Fetal bovine serum (FBS), penicillin, and streptomycin were purchased from Gibco (Carlsbad, CA, USA).

Samples from a BNC commercial sheet facial mask were used in the mechanical and moisture uptake assays, for comparison purposes. The BNC commercial sheet facial mask (Superstart Probiotic Boost Skin Renewal Biocellulose Mask, Elizabeth Arden, New York, NY, USA) is described as being derived from natural coconut water and embedded in a cosmetic formulation with skin renewal properties containing, among other ingredients, probiotics (*Lactococcus* ferment lysate, *Lactobacillus*), plant extracts (*Althae rosea* flower extract, *Polymnia sonchifolia* root juice, *Crithmum maritimum* extract), caffein, and humectants, such as glycerol, hyaluronic acid, or polyethylene glycol (PEG)-450. Samples of the BNC-commercial sheet were dried in a ventilated oven at 35 °C before use.

Fresh *E. globulus* Labill. leaves were collected from adult trees from an industrial plantation of The Navigator Company near Aveiro at Sever do Vouga (Braçal) (Aveiro, Portugal) and submitted to a hydro-distillation process until complete extraction of the essential oil fraction (2–3 h) using a modified Clevenger-type apparatus. At the end, an aqueous extract, the HDE, was recovered and lyophilized, stored, and protected from light in a desiccator, at room temperature, until use [40].

### 2.2. Total Phenolic Content of HDE

Total phenolic content (TPC) of HDE was determined using the Folin–Ciocalteu method, as described elsewhere [41], with some modifications. Briefly, in a 96-well plate, 150 μL of Folin–Ciocalteu reagent previously diluted (1:10, *v*/*v*) with water and 120 μL of aqueous sodium carbonate solution (75 g L^−1^) were added to 30 μL of an aqueous HDE solution in concentrations ranging from 1.95 μg to 500 μg mL^−1^ of extract. The reaction mixtures were incubated in the dark at room temperature during 60 min. After the incubation period, the absorbance was measured at 760 nm against a blank (water instead of extract) in a Thermo Scientific Multiskan^TM^ FC microplate reader (Thermo Fisher Scientific Inc., Waltham, MA, USA). TPC was calculated as gallic acid equivalents (GAE) from the calibration curve of gallic acid standard solutions (5.13–205.0 μg mL^−1^) and expressed as mg of GAE g^−1^ of lyophilized extract. The assay was performed five times, and each sample was analyzed in triplicate.

### 2.3. Production of the BNC Membranes

BNC membranes were produced in our laboratory using Hestrin–Schramm (HS) liquid culture medium (20 g L^−1^ glucose, 5 g L^−1^ peptone, 5 g L^−1^ yeast extract, 2.7 g L^−1^ disodium hydrogen phosphate, 1.15 g L^−1^ citric acid, pH 5) inoculated with the acetic acid bacteria *Gluconacetobacter sacchari* under static culture conditions [42]. After 4–6 days of culture at 30 °C, the BNC membranes were harvested and treated twice with 0.5 M NaOH at 80 °C for 30 min. Afterwards, membranes were washed several times with distilled water to eliminate the remaining medium components and bacterial cells. Finally, BNC membranes were whitened with a 1% sodium hypochlorite aqueous solution followed by repeated washes with distilled water until neutral pH was reached. Purified membranes were stored in ultrapure water at +4 °C until use.

### 2.4. Preparation of the BNC-G-HDE Membranes

Wet BNC membranes (diameter: ca. 7.0 ± 0.5 cm; thickness: 7000 ± 1000 μm) were loaded with different doses of HDE (expressed as mass of HDE per surface area of the membrane) and glycerol (7.5 mg cm^−2^) (Table 1), using the impregnation method. Succinctly, wet BNC membranes were weighted (about 180 mg of dry BNC) and drained by hand-pressing with laboratory grade absorbent paper until water content decreased to nearly 40% (estimated by weight loss). Then, membranes were soaked in 5 mL of an aqueous solutions containing the respective doses of both HDE and glycerol and maintained at room temperature until complete absorption of the HDE-G solution. Finally, BNC membranes were dried (in petri dishes) at 35 °C in a ventilated oven (Venticell Eco line, MMM group, Planegg, Germany) for at least 20 h. Dried membranes were stored and protected from the light in a desiccator at room temperature until use. For comparison purposes, pure BNC membranes (without the extract and glycerol) were dried as previously described, and membranes loaded with glycerol (BNC-G) were prepared following the same procedure.

### 2.5. Characterization of Membranes

#### 2.5.1. Thickness

The thickness of dry membranes was measured in five random sites using a hand-held digital micrometer (Mitutoyo Corporation, Tokyo, Japan) with an accuracy of 1 μm.

#### 2.5.2. Morphology

The surface and cross-section (cryo-fractured) morphologies of the membranes were analyzed by scanning electron microscopy (SEM). Micrographs were obtained using a high-voltage microscope (HR-FESEM SU 70 Hitachi, Tokyo, Japan) operated at 4.0 kV. Prior to image acquisition, samples were placed on a steel support and coated with carbon.

#### 2.5.3. Mechanical Performance

The mechanical performance of membranes in dry and wet (80% moisture) states was evaluated by tensile tests. The moisture content of wet membranes was defined according to the moisture content determined in the commercial BNC. Tensile tests were performed on a uniaxial Instron 5564 testing machine (Instron Corporation, Norwood, MA, USA) in the traction mode at a crosshead velocity of 10 mm min^−1^ using a 500 N static load cell. All measurements were conducted in at least five replicates using rectangular test specimens (5 × 1 cm^2^) and a gauge length of 30 mm. Stress (MPa) and strain (%) curves were plotted, and the Young’s modulus, tensile strength, and the elongation at break were determined using Instron BlueHill 3 software. In these assays, specimens of a commercial BNC facial mask were also tested in the same conditions, for comparison.

#### 2.5.4. Thermal Stability

Thermogravimetric analysis (TGA) was carried out with a SETSYS Setaram TGA analyzer (SETARAM Instrumentation, Lyon, France) equipped with a platinum cell. The samples were heated from room temperature to 800 °C at a constant rate of 10 °C min^−1^ under nitrogen atmosphere.

#### 2.5.5. Moisture Uptake Capacity

The moisture uptake capacity of all membranes was evaluated by placing dried specimens (2 × 2 cm^2^) of each membrane in a desiccator, at room temperature, with a relative humidity at ca. 52% using a saturated magnesium nitrate aqueous solution (52.89 ± 0.22% at 25 °C) [43]. The specimens were taken from the desiccator and weighed after 0.5 h, 1 h, 2.5 h, 24 h, and 48 h. All membranes were tested in triplicate. Moisture uptake was calculated as follows:

$$\mathrm{Moisture}\;\mathrm{uptake}\;\left(\%\right)=\left(W_{w}-W_{0}\right)\times W_{0}^{-1}\times 100$$

where W_0_ is the specimen initial weight, and W_w_ is the weight at each time point.

### 2.6. In Chemico Antioxidant Activity

The antioxidant activity of the HDE-loaded membranes was estimated using the DPPH free radical scavenging method, following as previously reported procedure [44], with some modifications. The reaction is based on the decrease in the absorbance of the DPPH solution resulting from the radical scavenging by the antioxidant compounds, with consequent change in color of the DPPH solution from purple to pale yellow [45]. Briefly, specimens (2 × 5 cm^2^) of each membrane were added to 3.75 mL of ethanol (EtOH) and phosphate-buffered saline (PBS) solution (60:40; pH 5.5). Then, 250 μL of DPPH (1 mM) solution in ethanol was added, and the resulting mixtures were incubated in the dark with gentle mixing (100 rpm) at 22 °C for 0.5 h, 1 h, and 2.5 h, and then the absorbance at 517 nm was read against the blank (EtOH:PBS 1× pH 5.5) using a Multiskan^TM^ FC microplate reader (Thermo Scientific, Waltham, Massachusets, EUA). For comparison, reaction mixtures containing the HDE diluted in EtOH:PBS 1× pH 5.5 at a final concentration equivalent to the maximum quantity of HDE that could be released from each membrane (HDE 1: 2.5 μg mL^−1^; HDE 1.5: 3.75 μg mL^−1^; HDE 2: 5.0 μg mL^−1^; HDE 3: 7.5 μg mL^−1^) were also included in the assay. A control consisting of EtOH:PBS 1× pH 5.5 with DPPH and without membrane was used. Three independent assays were carried out for each sample. The DPPH radical scavenging activity was calculated from the absorbance of each sample (A_sample_) with respect to the control DPPH absorbance (A_DPPH_) as follows:

$$\mathrm{DPPH}\;\mathrm{scavenging}\;\mathrm{activity}=\left(A_{\mathrm{DPPH}}-A_{\mathrm{sample}}\right)\times A_{\mathrm{DPPH}}^{-1}\times 100$$

### 2.7. Evaluation of the Stability of the BNC-G-HDE2 Membrane under Storage

Based on the “Guidelines on stability testing for cosmetic products” [46], the storage stability of BNC-G-HDE2 was evaluated. For this, samples (14 cm^2^) of BNC-G (as control) and BNC-G-HDE2 membranes were placed in glass vials and stored for 3 months in the dark at the expected normal storage condition, at room temperature (22–25 °C) and 52% RH (condition I, using a magnesium nitrate saturated solution [43]) and at accelerated condition of 40 °C and 75% RH (condition II, using a sodium chloride saturated solution [43]). The relative humidity was periodically monitored using a thermohydrometer to ensure constant humidity during the entire storage period. All samples were analyzed before and after 1, 2, and 3 months of storage regarding their antioxidant activity (as described in Section 2.6). The macroscopic aspect of membranes was also recorded. For each condition, all samples were analyzed in triplicate.

### 2.8. In Vitro Biological Assays

#### 2.8.1. Cell Culture

Human keratinocytes (HaCaT, from CLS, Cell Lines Service, Eppelheim, Germany) and mouse fibroblasts (NIH/3T3, ATCC CRL-1658, Manassas, VA, USA) cell lines were cultured using Dulbecco’s Modified Eagle’s Medium (DMEM), at 37 °C, in a humidified 5% CO_2_–95% air atmosphere. The medium was supplemented with 10% (*v*/*v*) heat-inactivated fetal bovine serum (FBS), 1% (*v*/*v*) pen/strep, 3.7 g L^−1^ sodium bicarbonate, and 1 mM sodium pyruvate. Cells were detached with trypsin-EDTA solution 1×.

#### 2.8.2. Cytotoxicity Assay

The cytotoxicity of the membranes was evaluated using MTT reduction assay. Samples (2.5 cm^2^) of the membranes (BNC-G, BNC-G-HDE1, BNC-G-HDE1.5, BNC-G-HDE2, BNC-G-HDE3) were sterilized twice by UV radiation on each side during 20 min and further incubated, for 24 h, in 2.5 mL of complete DMEM medium at 37 °C in a humidified 5% CO_2_–95% air atmosphere, to prepare each membrane extract.

Meanwhile, 2 × 10^4^ and 1 × 10^4^ cells/well were seeded in 96-well plates for HaCaT and NIH/3T3 cells, respectively. After 24 h, the same volume of the membrane extract was used to replace the culture medium and cells were further incubated for 24 h at 37 °C. As control, cells were treated in the same way as described for samples but exposed only to DMEM medium. After 24 h, the medium was removed, and the cytotoxicity determined as previously described [47]. Briefly, a fresh solution of MTT (0.5 mg L^−1^) prepared in Krebs medium (pH 7.4) was added and incubated at 37 °C during 2 h (HaCaT cells) or 4 h (NIH/3T3 cells). After that, the MTT solution was replaced by DMSO and incubated for 10 min, with shaking, to completely dissolve the formazan crystals. After incubation, the absorbance was measured at 570 nm in a spectrophotometer (SLT spectra II). The results of 3 independent experiments, with 3 replicates each, were expressed as percentage (%) of the absorbance value obtained in control and graphically presented as % of MTT reduction.

#### 2.8.3. Anti-Senescence Activity

The anti-senescence activity of the BNC-G-HDE2 membrane was evaluated using the β-galactosidase (β-gal) staining assay. The BNC-G membrane was used as control. To prepare the membranes extracts, sterilized samples (10 cm^2^) were incubated in 10 mL of complete DMEM medium as previously described for the cytotoxicity assay.

NIH/3T3 cells were seeded in 12-well plates at a density of 2.5 × 10^4^ cells/well and allowed to stabilize for 24 h. Subsequently, 12.5 µM etoposide was used to induce cellular senescence in NIH/3T3 for 24 h. Etoposide-stimulated cells were then treated with the BNC-G-HDE2 membrane extract for another 24 h. As control, non-treated cells were incubated with DMEM medium or with extracts of BNC-G and BNC-G-HDE membranes. After incubation, culture medium was removed, and cells were washed with PBS and marked with β-gal solution prepared as described by the manufacturer (Cell Signaling Technology, Danvers, MA, USA). The analysis of the positive cells for senescence was performed at 20× magnification using a widefield microscope (Carl Zeiss, Oberkochen, Germany). At least 3 independent experiments were performed in replicate, and the percentage of β-gal-positive cells was determined using four microscopic images.

### 2.9. Statistical Analysis

In the treatment of the results from the characterization, antioxidant activity, and storage stability test of the membranes, a one-way analysis of variance (ANOVA) followed by Tukey’s test was used to assess the level of significance. The results are expressed as the mean ± standard deviation of the mean. Regarding in vitro assays, the normality of the data distribution was assessed by the D’Agostino–Pearson and Shapiro–Wilk normality tests. Statistical comparisons between groups were performed by ANOVA followed by Dunnett’s post-hoc test or unpaired Students’s *t*-test. The results are presented as the mean ± standard error of the mean of the indicated number of experiments.

Significance was accepted at values of *p* < 0.05. All statistical calculations were performed using GraphPad Prism software (8.0.2, GraphPad Software Inc., San Diego, CA, USA).

## 3. Results and Discussion

**Figure 1:**
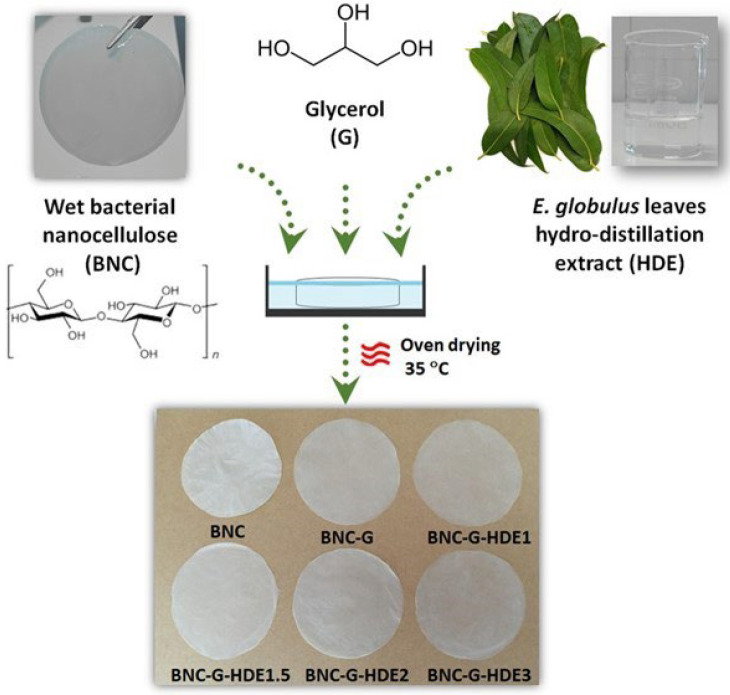
Scheme showing the preparation of the bacterial nanocellulose (BNC) membranes loaded with glycerol (G) (7.5 mg cm^−2^) and different doses (1–3 μg cm^−2^) of the hydro-distillation extract (HDE) of *E. globulus* leaves, using the impregnation method, followed by drying in a ventilated oven at 35 °C. The macroscopic aspect of the resulting membranes is also shown.

Bioactive BNC membranes, incorporating different doses of an aqueous extract obtained from the hydro-distillation of *E. globulus* leaves, were prepared, envisioning their application as anti-aging skin care sheet masks. Four BNC membranes containing 7.5 mg cm^−2^ of glycerol (acting as plasticizer and humectant) [39,48] and different contents of the HDE, namely 1.0 μg cm^−2^ (BNC-G-HDE1), 1.5 μg cm^−2^ (BNC-G-HDE1.5), 2.0 μg cm^−2^ (BNC-G-HDE2), and 3.0 μg cm^−2^ (BNC-G-HDE3), were prepared (Table 1). A pure BNC membrane (BNC) and a BNC membrane with only glycerol (BNC-G) were also prepared for comparison (Table 1). The BNC-G-HDE membranes were fabricated by the simple impregnation method, based on the diffusion of the HDE aqueous solutions and glycerol into the hydrophilic and highly porous BNC network (Figure 1), according to previous studies that described this process for the incorporation of a mixture of plant extracts [13] and caffein [23].

With the addition of the HDE and glycerol, the thickness of the dry BNC membranes increased from 50 ± 1 μm for the pure BNC to 87 ± 4 μm for the BNC-G, and to values in the range of 79 ± 4 μm to 98 ± 8 μm for membranes loaded with HDE and glycerol, which indicates the successful incorporation of the components of the extract and glycerol into the 3D structure of BNC. The macroscopic aspect of the obtained membranes (Figure 1) is indicative of a homogeneous distribution of the HDE and glycerol within the nanostructure of BNC. It is also evident that the inclusion of glycerol turns the membranes more translucent, as previously described [20]. Moreover, the incorporation of the HDE did not result in visible color alterations of the whitish BNC membranes, which is in line with the colorless appearance of the HDE aqueous solution (Figure 1).

All the obtained BNC membranes were characterized in terms of morphology, mechanical performance, thermal stability, and moisture uptake capacity. Furthermore, the antioxidant activity and the in vitro cytotoxicity of the membranes were also assessed. For the BNC-G-HDE2 membrane (2.0 μg cm^−2^), the stability of the antioxidant activity after 3 months of storage at 22–25 °C and 52% RH or at 40 °C and 75% RH as well as the in vitro anti-senescence activity were also evaluated.

### 3.1. Morphology

**Figure 2:**
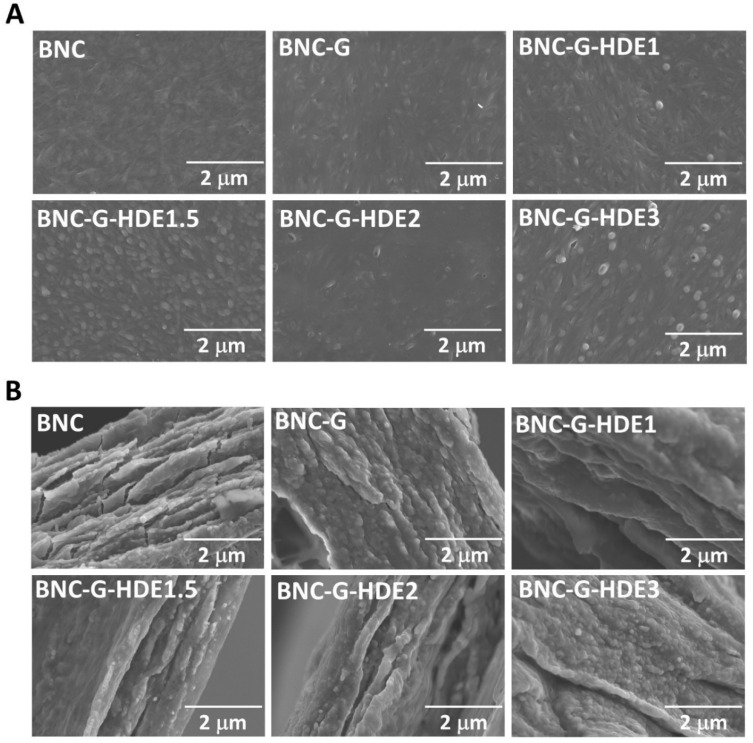
(**A**) Surface and (**B**) cross-section SEM micrographs (×20 k magnification) of the membranes of BNC, BNC-G, and BNC-G with different doses of HDE.

The morphology of the BNC, BNC-G, and BNC-G-HDE membranes was evaluated by SEM analysis, as depicted in Figure 2. In the surface micrograph of the pure BNC, its characteristic 3D nanofibrillar network is clearly noticeable; however, this microstructure is less perceivable on the BNC-G and BNC-G-HDE membranes, especially with the increment of the amount of extract, which, together with glycerol, covers the cellulose nanofibrils. This effect was already observed in BNC membranes incorporating only glycerol [49] or glycerol and active pharmaceutical ingredients (e.g., caffein, diclofenac, and lidocaine) [22]. It can also be seen that these membranes display a less compact structure than the pristine BNC due to the entrapment of glycerol (as well as the extract components) in the BNC network. The incorporation of glycerol molecules between BNC nanofibrils reduces the intermolecular hydrogen bonds between cellulose nanofibrils [49], and consequently, diminishes the collapse of the BNC structure during drying, resulting thereby in membranes with increased flexibility [23]. In the cross-section images, the filling of the lamellar spaces of BNC with glycerol and the extract is also clearly observed. Similar results were also reported for BNC-based materials loaded with an ethanolic solution of propolis extract [50], *Scrophularia striata* Boiss extract [51], or hyaluronic acid [52]. Thus, these results confirm the successful incorporation of glycerol and HDE into the BNC membranes, with no evident extract agglomerates formation. This observation is an indication of the good compatibility of the extract components (phenolic compounds) with the cellulose fibrils and glycerol.

### 3.2. Mechanical Performance

The mechanical performance of all BNC membranes was evaluated by tensile tests and compared with that of a commercial BNC-based facial mask. Considering that a sheet facial mask is typically applied in the wet state, the mechanical performance of the prepared membranes was evaluated in both dry and wet (80% moisture content) states. The evaluation of the resistance and elasticity of a sheet facial mask is fundamental because it must be sufficiently resistant to be handled and, at same time, elastic enough to enable a perfect fit and tight adherence to the skin, thereby allowing a better penetration of the active compounds into the skin [13].

**Figure 3:**
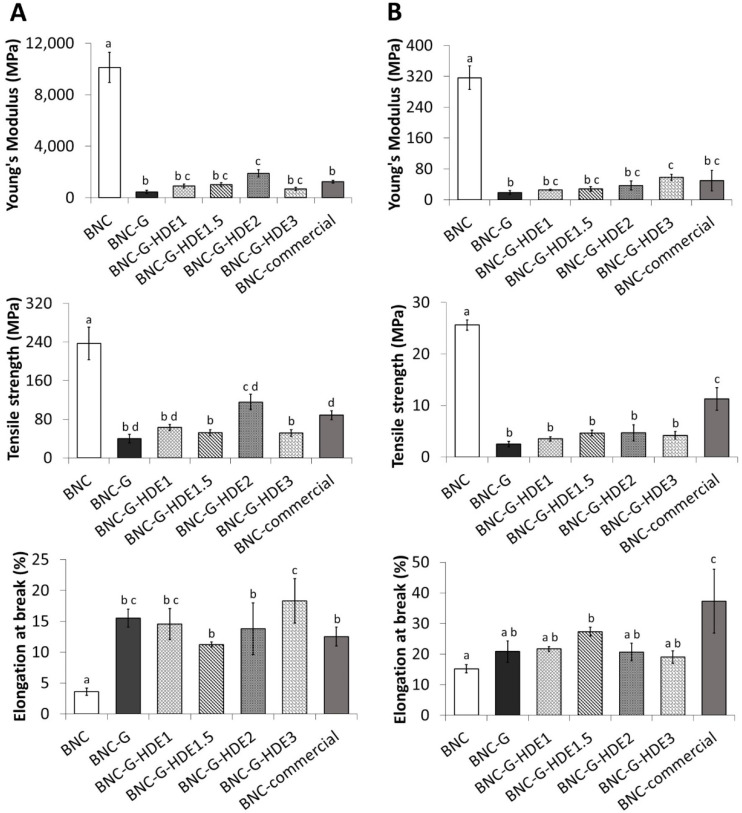
Mechanical properties (Young’s modulus (MPa), tensile strength (MPa), and elongation at break (%)) evaluated by tensile tests of the (**A**) dry and (**B**) wet membranes: BNC, BNC-G, and BNC-G with different doses of HDE and BNC from a commercial facial mask (BNC-commercial). The values are the mean of five replicates, and the errors bars correspond to standard deviations. Means with different letters indicate a significant difference (*p* < 0.05).

The results of the tensile tests of the dry membranes (Figure 3A) show that the incorporation of glycerol significantly affected the mechanical properties of the BNC membranes by improving, to a high extent, their elasticity. This is evident by the significant increase in the value of the elongation at break from 3.6 ± 0.6% for BNC to 15.5 ± 1.4% for BNC-G, accompanied by a significant decrease in the Young’s modulus (from 10.1 ± 1.2 GPa for BNC to 0.5 ± 0.1 GPa for BNC-G) and in the tensile strength (from 237 ± 34 MPa for BNC to 40 ± 9 MPa for BNC-G). These results agree with previous studies regarding BNC-based materials incorporating glycerol as plasticizer [39,49,53]. As explained above, the incorporation of glycerol into the BNC network causes the reduction of the intermolecular forces between the cellulose nanofibrils, thereby increasing the inter-fiber space and their mobility, resulting in more elastic membranes [49]. Regarding the mechanical behavior of the wet membranes (Figure 3B), the effect of the addition of glycerol on the elongation of the membranes is not so obvious. In fact, BNC shows an identical elongation value to that of BNC-G (BNC: 15.2 ± 1.3%, BNC-G: 20.8 ± 3.5%), which may be associated with the presence of water molecules in the BNC network that will also weaken the interfibrillar bonds [24], making the wet BNC more stretchable. However, as in the dry membranes, a significant reduction in the Young’s modulus (BNC: 0.3 ± 0.0 GPa vs. BNC-G: 0.02 ± 0.01 GPa) and in tensile strength (BNC: 26 ± 1 MPa vs. BNC-G: 2 ± 1 MPa) is observed after glycerol loading.

The incorporation of the HDE into the BNC membranes does not alter, in a significant way, most of the mechanical properties of the dry membranes when compared with the BNC-G counterpart. The exception is the increase of tensile strength and Young’s modulus for BNC-G-HDE2 and the increase of the elongation at break of BNC-G-HDE3. All BNC-G-HDE membranes show high elongations (11–18%) together with small values of Young’s modulus (0.7–1.9 GPa) and tensile strength values between 51 and 116 MPa. Similarly, in wet state, it was also confirmed that the addition of HDE does not significantly influence most of the mechanical properties of BNC-G (Figure 3B). The wet BNC-G-HDE membranes also show high elongations (19–27%), low values of Young’s modulus (0.03–0.06 GPa), and tensile strength values between 4 and 5 MPa.

Taken all together, the mechanical properties discussed above confirm that the BNC-G-HDE membranes are pliable enough to be manipulated and moldable to the skin, as is desirable for the intended application. In fact, the results demonstrate that these membranes are really promising, especially considering the comparison with the commercial BNC mask. The dry BNC-G-HDE membranes have identical or even significantly (*p* < 0.05) superior mechanical properties (e.g., elongation at break of dry BNC-G-HDE3) to those of the commercial BNC counterpart When evaluated in wet state, the commercial BNC shows higher values of elongation at break and tensile strength than those observed in the BNC-G-HDE membranes, whereas values of the Young’s modulus are identical. Differences in the content of plasticizers and humectants (e.g., glycerol, hyaluronic acid, PEG-450) can explain the increased elasticity of wet commercial BNC compared to the BNC-G-HDE membranes developed in the present study.

### 3.3. Thermal Stability

**Figure 4:**
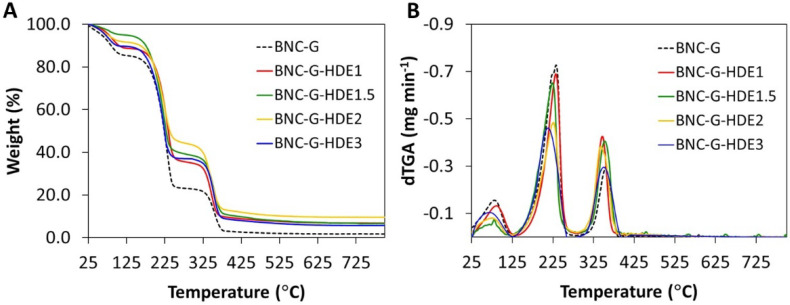
(**A**) Thermogravimetric curves and (**B**) the respective derivatives of BNC-G and BNC-G loaded with different doses of HDE heated from room temperature up to 800 °C (10 °C min^−1^) under inert (N_2_) atmosphere.

For insight into the thermal stability of the BNC-G-HDE dry membranes, thermogravimetric analysis was performed under inert (N_2_) atmosphere. The BNC-G membrane was analyzed as control. The degradation profiles and corresponding derivatives are depicted in Figure 4. All membranes show an initial weight loss below 100 °C, credited to the evaporation of adsorbed water from the polymeric matrix, which confirms the hydrophilicity of BNC and the hygroscopic nature of glycerol. Apart from this dehydration step, all membranes display a two-step weight loss profile. The thermal profile of the BNC-G membrane shows a step of weight loss with initial and maximum decomposition temperatures of about 125 °C and 233 °C, respectively, mainly associated with the degradation of glycerol, in good agreement with previous results obtained elsewhere with plasticized BNC [49]. The second weight loss stage occurs at initial and maximum decomposition temperatures of about 295 °C and 355 °C, respectively, and is attributed to the decomposition of the cellulose glycosyl units [30].

Regarding the membranes incorporating the HDE, the weight loss step assigned to the decomposition of glycerol exhibits a maximum decomposition temperature at ca. 232 °C for BNC-G-HDE1, 224 °C for BNC-G-HDE1.5, 226 °C for BNC-G-HDE2, and 213 °C for BNC-G-HDE3. Similar to BNC-G, the second weight loss step is observed at maximum decomposition temperatures of about 355 °C for BNC-G-HDE1, 353 °C for BNC-G-HDE1.5, 343 °C for BNC-G-HDE2, and 350 °C for BNC-G-HDE3, owing to the decomposition of the BNC polymeric chains. From the thermograms, it was not possible to identify the weight loss attributed to the decomposition of the HDE phenolic compounds, which according to the literature may occur in the range of 100–300 °C [54,55]. However, given the low ratio (<0.1%, *w*/*w*) of the HDE in the BNC membranes, the weight loss associated with the extract components is probably not perceivable.

These results indicate that all membranes are stable up to ca. 125 °C, which is comparable with results previously reported for glycerol-plasticized BNC membranes [49] and is adequate for the intended use as a facial mask, considering its use at body temperature.

### 3.4. Moisture Uptake Capacity

**Figure 5:**
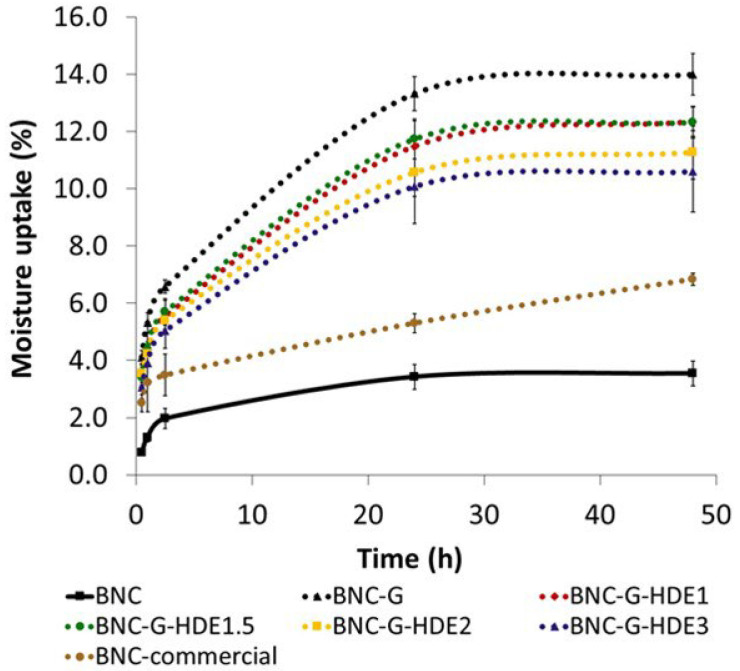
Moisture-uptake capacity of the membranes of BNC, BNC-G, and BNC-G loaded with different doses of HDE and BNC from a commercial facial mask (BNC-commercial) at room temperature and relative humidity (RH) ca. 52% (using a saturated magnesium nitrate aqueous solution). Moisture uptake (%) was calculated at 0.5 h, 1 h, 2.5 h, 24 h, and 48 h. The values are the mean of three replicates, and the errors bars correspond to standard deviations.

Moisture retention is a highly desirable property of sheet facial masks aiming to avoid the dehydration of the polymeric structure and ensure the adherence of the mask to skin during the entire time of treatment [24]. At the same time, the swelling of the nano-porous structure of BNC, due to water binding, will also facilitate the delivery of the incorporated active compounds. Thus, the moisture uptake capacity of the prepared dry membranes was determined to evaluate their ability to absorb environmental humidity. Therefore, membranes were placed in a desiccator with controlled humidity (RH ca. 52%) at room temperature, and moisture uptake was monitored over time, from 30 min up to 48 h (Figure 5). The RH of about 52% used in the assay is close to the value of 45 ± 5%, indicated as general guidelines to perform hydration studies with moisturizing cosmetics [56].

All the prepared BNC membranes can absorb environmental humidity, showing an incremental uptake over time and reaching a maximum at 48 h, which is in agreement with a previous work with BNC loaded with silk-sericin [24]. As expected, the incorporation of glycerol, a highly hygroscopic compound, resulted in BNC membranes with higher aptitude to uptake environmental humidity, even for short periods of time, such as 1 h, with BNC-G membranes absorbing 5.3 ± 0.3% of moisture after this time, and pristine BNC absorbing only 1.3 ± 0.2%. A similar behavior was observed for the BNC-G membranes loaded with the HDE, presenting a moisture uptake of 4.4 ± 0.6% for BNC-G-HDE1, 4.4 ± 0.3% for BNC-G-HDE1.5, 4.2 ± 0.3% for BNC-G-HDE2, and 3.9 ± 0.6% for BNC-G-HDE3 after 1 h. The amount of the HDE seems to have no influence in the moisture uptake capacity of the membranes, since the differences are not significant (*p* > 0.05). Considering that the time of treatment of a beauty mask is generally between 20 min and 1 h, the capacity of the BNC-G-HDE membranes to rapidly absorb environmental humidity is fundamental to guarantee the adherence of the BNC sheet to the skin during the entire treatment and favor the release of the active compounds. It is also noteworthy that for the short period of 1 h, the moisture uptake capacity of BNC-G-HDE membranes is identical (*p*> 0.05) to that obtained for the commercial BNC mask (3.2 ± 1.0%).

### 3.5. In Chemico Antioxidant Activity

The TPC of the *E. globulus* HDE, determined using the Folin–Ciocalteu method, accounted for 329 ± 9 mg of GAE g^−1^ of lyophilized extract. This result confirms the presence of a high content of phenolic compounds in this extract, being comparable with TPC values previously reported for phenolic extracts of *E. globulus* leaves (311 ± 20 mg GAE g^−1^ of dry extract) [57].

**Figure 6:**
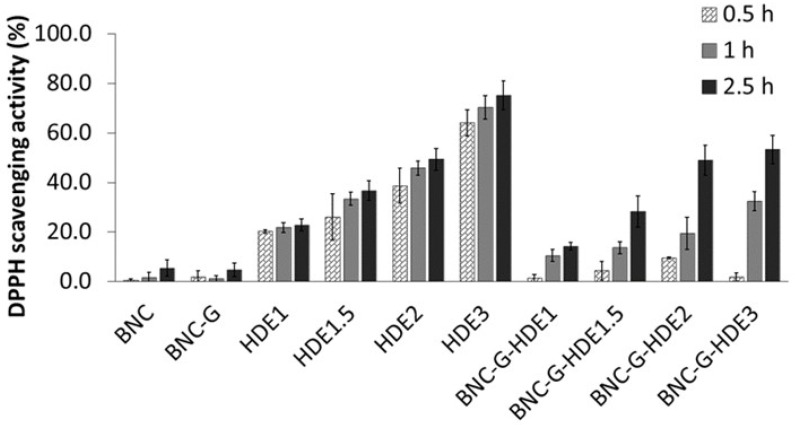
Antioxidant activity of the membranes of BNC, BNC-G, and BNC-G loaded with different doses of HDE over time (up to 2.5 h) assessed by the DPPH free radical scavenging assay in an ethanol and phosphate-buffered saline (60:40) solution, pH 5.5. Results are expressed as means of three independent assays, and error bars correspond to standard deviations.

Phenolic compounds are well known for their antioxidant properties resulting from the scavenging of free radicals, which is one of the mechanisms to prevent cellular oxidative stress [29]. Therefore, the antioxidant activities of the HDE and of the prepared BNC membranes (BNC, BNC-G, BNC-G-HDE1, BNC-G-HDE1.5, BNC-G-HDE2, and BNC-G-HDE3) were assessed by the DPPH free radical scavenging method (Figure 6). The antioxidant activity was monitored up to 2.5 h, based on the usually short duration of application of a facial mask and on procedures described elsewhere [44,58].

The pure BNC membrane does not present considerable antiradical scavenging activity, which agrees with data reported in previous studies [51,59]. Similar results were obtained for the BNC-G membranes since glycerol is not an antioxidant compound. Regarding the HDE aqueous solutions, they show a dose-dependent antioxidant activity, which can be attributed to their content in phenolics, similar to results previously reported for an aqueous *E. globulus* leaf extract [57]. The maximum DPPH scavenging activity was attained at the end of 2.5 h by the HDE3 solution (7.5 μg mL^−1^) (75.1 ± 5.8%). This result indicates that the HDE has a higher antioxidant activity when compared to an ethanolic extract of dried commercial *E. globulus* biomass, which only shows a DPPH scavenging activity of 65% (at 0.5 h incubation) for an extract concentration of 250 μg mL^−1^ [37]. Regarding the BNC-G-HDE membranes, the results revealed that all membranes display antioxidant activity with a dose-dependent response that increases over time. The maximum DPPH scavenging activities were reached at the end of 2.5 h, with 14.2 ± 1.6%, 28.1 ± 6.3%, 48.9 ± 6.1%, and 53.3 ± 5.7% for BNC-G-HDE1, BNC-G-HDE1.5, BNC-G-HDE2, and BNC-G-HDE3 membranes, respectively. Therefore, the results clearly show that the incorporation of the HDE into the BNC network successfully imparts the pure BNC with antioxidant activity, similar to what was achieved in previous works with extracts of *S. striata* Boiss [51] or *Epilobium angustifolium* L. [60].

These results also demonstrate that the HDE is gradually released from the membranes, and after 2.5 h, the DPPH scavenging activities obtained for all membranes, except for BNC-G-HD3, are identical (*p* > 0.05) to those achieved for the corresponding HDE solution. The difference (*p* < 0.05) between BNC-G-HDE3 and HDE3 samples may be related to the kinetics of the HDE release from the membrane. These are promising results regarding the use of these membranes for the delivery of the active compounds of the HDE. In particular, the BNC-G-HDE2 membrane seems preferable for further studies, because it shows similar results, in terms of antioxidant activity, to the membrane with highest dose of HDE, even though a lower dose of HDE is used, which is especially relevant when future scale-up is considered.

### 3.6. Evaluation of the Stability of the BNC-G-HDE2 Membrane under Storage

Environmental factors (e.g., temperature, light, and oxygen) are known to have an impact on the structure stability of phenolic compounds and on their antioxidant activity [61]. The storage temperature and light are among the most reported factors to be associated with the reduction of antioxidant activity of extracts containing phenolic compounds [62,63]. Hence, based on international guidelines on stability testing of cosmetic products [46], an exploratory stability test was performed to evaluate the changes in the antioxidant activity of the BNC-G-HDE2 membrane under normal storage conditions, namely at 22–25 °C and 52% RH (condition I) and at accelerated conditions (viz. exaggerated storage conditions), namely at 40 °C and 75% RH (condition II) for 3 months.

**Figure 7:**
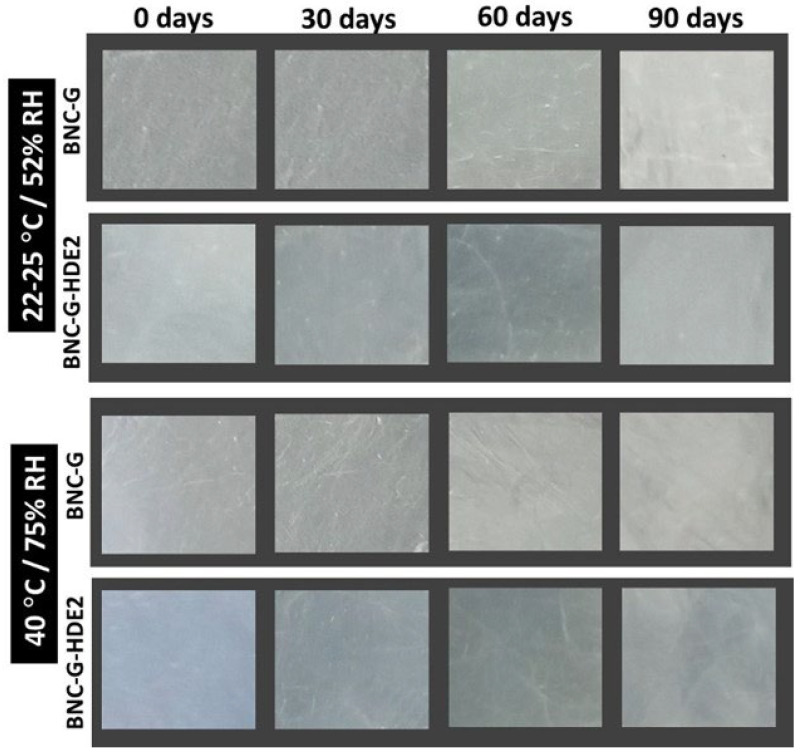
Photographs of the macroscopic aspect of the BNC-G and BNC-G-HDE2 membranes upon 3 months of storage at expected normal storage (condition I: 22–25 °C/52% RH) and accelerated conditions (condition II: 40 °C/75% RH).

Observation of the macroscopic aspect of the membranes (Figure 7) shows that there are no significant alterations in their homogeneity over the 3 months of storage at both storage conditions.

**Figure 8:**
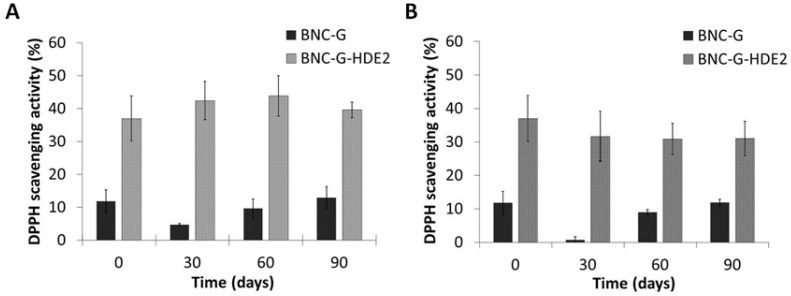
Antioxidant activity assessed by the DPPH free radical scavenging assay in ethanol and phosphate-buffered saline solution (60:40; pH 5.5) of the BNC-G and BNC-G-HDE2 membranes over 3 months of storage at (**A**) expected normal storage (condition I: 22–25 °C/52% RH) and (**B**) accelerated conditions (condition II: 40 °C/75% RH). Results are expressed as means of three independent assays, and error bars correspond to standard deviations.

Results from the antioxidant activity of the BNC-G (control) and BNC-G-HDE2 membranes over the 3 months of storage in each condition are depicted in Figure 8. As in the assay of the antioxidant activity of BNC-G-HDE membranes (Section 3.5), the values obtained for the antioxidant activity of BNC-G are not significant. Regarding the BNC-G-HDE2 membrane, when it is stored at 22–25 °C and 52% RH (Figure 8A), the antioxidant activity is kept unchanged over the entire assay (*p* > 0.05), showing values of 37.0 ± 6.9% in the beginning of the assay and 39.6 ± 2.4% after 3 months. Similar results were obtained after storage at accelerated conditions (Figure 8B) of 40 °C and 75% RH, in which the variation in the antioxidant activity after 1 month (31.7 ± 7.5%) and after 3 months (31.0 ± 5.2%) is not significantly different (*p* > 0.05) from that at the beginning of the assay. Hence, these results indicate that the antioxidant activity of BNC-G-HDE2 is stable for at least 3 months under these storage conditions, meaning that no apparent degradation of the phenolic compounds from the HDE occurs within the membranes. Moreover, with these promising results under accelerated conditions, it is predictable that the stability of membranes will be longer than 3 months under normal storage conditions.

### 3.7. In Vitro Biological Assays

#### 3.7.1. Cytotoxicity Assay

The evaluation of the safety of the BNC-based membranes is essential for their application as facial masks since this implies their direct contact with skin. Therefore, the cytotoxicity of the BNC-G (for control) and the four BNC-G-HDE membranes was evaluated using the MTT assay, with extracts of the membranes, towards human keratinocytes (HaCaT) and mouse fibroblasts (NIH/3T3) cells. These cell lines were selected for being representative of the two important structural cell types from skin. Keratinocytes are the main cells of the epidermis (the uppermost skin layer), while fibroblasts are part of the dermis (second skin layer) [64].

**Figure 9:**
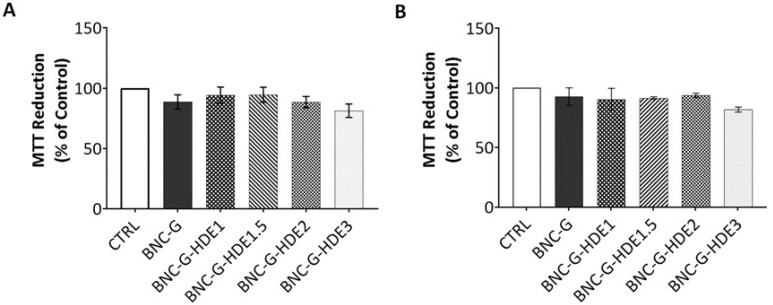
Cell viability assessed by theMTT reduction assay of (**A**) NIH/3T3 and (**B**) HaCaT cells after 24 h of exposure to extracts of the membranes of BNC-G and BNC-G loaded with different doses of HDE. As control, cells were treated in the same way as samples but exposed only to Dulbecco’s Modified Eagle’s Medium (DMEM). Results are expressed as means of three independent assays, and error bars correspond to standard error mean.

The results obtained for the BNC-G membrane (Figure 9) are in line with the well-known non-cytotoxicity of pure BNC towards HaCaT [52,65] and NIH/3T3 [65,66] cells, and of BNC plasticized with glycerol on HaCaT cells [39]. Concerning the results of the cytotoxicity of BNC membranes loaded with the HDE, it is noticeable that, independently of the HDE dose, the membranes have no significant effect on the viability of either HaCaT or NIH/3T3 cells. All values of cell viability obtained for BNC-G-HDE membranes are above 70% and are therefore considered non-cytotoxic towards these cell lines, according to the international standard ISO 10993-5 for the biological evaluation of medical devices (Part 5: Tests for in vitro cytotoxicity). Thus, these results are a confirmation that the incorporation of these doses of the *E. globulus* HDE into BNC is completely safe for dermal applications. Indeed, these data are in agreement with results reported elsewhere showing the non-cytotoxicity of an ethanolic extract from *E. globulus* biomass (1, 10, and 100 μg mL^−1^) tested on normal human dermal fibroblasts [37].

#### 3.7.2. Anti-Senescence Activity

The skin aging process has been associated not only with alterations in the composition and structural integrity of the dermal extra cellular matrix (ECM) [67] but also the increase of senescent cells in the dermal layer [68]. The accumulation of senescent dermal fibroblasts is now recognized as playing an active role in the skin aging process, contributing to the decline of the tissue functionality [69,70].

In this sense, the anti-senescence activity of the BNC-G-HDE2 membrane was assessed towards mouse fibroblasts (NIH/3T3 cell line). The BNC-G-HDE2 membrane was selected for this evaluation because, together with BNC-G-HDE3, it shows one of the highest antioxidant activities, and since a lower quantity of HDE is used in the BNC-G-HDE2, it is preferable with regard to a future economic application.

**Figure 10:**
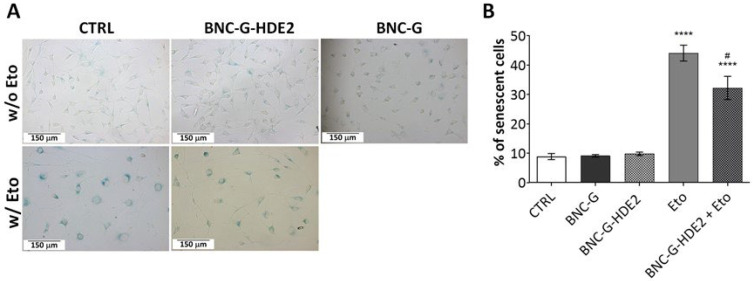
Anti-senescent effect of the BNC-G-HDE2 membrane extract on etoposide (Eto)-stimulated NIH/3T3 fibroblasts after 24 h exposure, assessed through the quantification of senescence-associated β-galactosidase (β-gal). As control, cells not treated with Eto were incubated with DMEM (CTRL) or with extracts of BNC-G and BNC-G-HDE membranes. (**A**) Representative images were acquired after staining under a widefield microscope at 20× magnification. (**B**) The percentage of senescent cells was determined. Results represent the mean of at least 3 independent assays performed in replicate, and error bars correspond to standard error mean. **** denotes statistical differences (*p* < 0.05) against CTRL, and # denotes statistical difference (*p* < 0.05) between Eto and BNC-G-HDE2 + Eto.

Cellular senescence was induced with the genotoxic agent etoposide, and the activity of β-gal, a broadly used biomarker for senescent cells [71], was measured. According to the data presented in Figure 10, neither BNC-G nor BNC-G-HDE2 membranes activate senescence-associated β-gal of NIH/3T3 cells. On the contrary, upon etoposide treatment, the number of senescence-associated β-gal-positive (blue staining) NIH/3T3 cells significantly increases compared to control (44.1 ± 2.7% versus 8.9 ± 1.0%). This is also visible in the acquired morphology of the NIH/3T3 cells, showing the characteristic senescent phenotype with enlarged and flattened cells (Figure 10A). Noticeably, after 24 h of treatment with the BNC-G-HDE2 extract, there is a significant reduction in the number of senescent NIH/3T3 cells (32.2 ± 4.0%) (Figure 10B). This positive effect is surely credited to the components of the HDE, namely phenolics compounds that are released from the membrane. In fact, phenolic compounds have already been demonstrated to have beneficial effects on preventing cellular senescence by suppressing the senescence-associated β-gal, mostly due to their known role as inhibitors of oxidative damage [72,73]. Other examples in line with the results obtained here are the findings on the effects of the *Sonchus oleraceus* L. leaf extract (containing caftaric acid, chlorogenic acid, and chicoric acid) on H_2_O_2_-induced cell senescence in WI-38 human lung diploid fibroblast cells [74], or of olive phenols (hydroxytyrosol and oleuropein) on pre-senescent human dermal and lung fibroblasts [72]. However, as far as we know, this is the first time that this activity has been reported for BNC membranes enriched with plant extracts. Therefore, these are promising results suggesting the considerable potential of the developed BNC-G-HDE membranes for anti-aging skin care applications.

## 4. Conclusions

Aiming at the development of an entirely bio-based material with bioactive and adequate mechanical properties for potential anti-aging skin care applications, BNC membranes loaded with glycerol and different doses (1–3 μg cm^−2^) of an aqueous *E. globulus* extract derived from hydro-distillation (HDE) of leaves containing phenolic compounds (329 ± 9 mg GAE g^−1^ of lyophilized extract) were prepared through the impregnation method. All BNC-G-HDE membranes exhibited a resistant but still elastic mechanical behavior, similar (at both dry and wet states) to that of a commercial BNC facial mask. The ability of the BNC-G-HDE membranes to uptake environmental moisture was higher than that of pure BNC and also comparable to that of the commercial BNC mask. BNC-G-HDE membranes were found to be thermally stable up to about 125 °C. Moreover, the incorporation of the HDE confers a dose-dependent antioxidant activity (DPPH radical scavenging assay) to BNC. The stability of the antioxidant activity of the membrane with 2 μg cm^−2^ of HDE was proven after storage over 3 months at expected normal (22–25 °C/52% RH) and accelerated (40 °C/75% RH) storage conditions. All BNC-G-HDE membranes were shown to be non-cytotoxic towards HaCaT and NIH/3T3 cell lines. Moreover, the treatment with BNC-G-HDE2 membrane was demonstrated to cause a reduction in the percentage of senescent cells (ca. 32%) compared to that observed in treated cells only with etoposide (ca. 44%). The findings reported here clearly highlight the potential of the prepared HDE-loaded BNC membranes as robust sheet facial masks for anti-aging skin care.

## Figures and Tables

**Table 1:** List of the prepared BNC membranes with the respective content of HDE and glycerol (relative to the surface area of the membrane), and the corresponding thickness values.

Membrane	Glycerol (mg cm^−2^)	HDE Dose (μg cm^−2^)	Thickness (μm)
BNC	-	-	50 ± 1
BNC-G	7.5	-	87 ± 4
BNC-G-HDE1	7.5	1.0	96 ± 5
BNC-G-HDE1.5	7.5	1.5	98 ± 8
BNC-G-HDE2	7.5	2.0	79 ± 4
BNC-G-HDE3	7.5	3.0	89 ± 7
